# The sudden transition to remote learning in response to COVID-19: lessons from Malaysia

**DOI:** 10.1057/s41599-023-01751-6

**Published:** 2023-05-20

**Authors:** Mohd Idzwan Mohd Salleh, Nor Aziah Alias, Suriyani Ariffin, Zainuddin Ibrahim, Ahmad Razi Ramli, Sharifah Aliman

**Affiliations:** 1 College of Computing, Informatics and Media, Universiti Teknologi MARA, Puncak Perdana, Selangor Malaysia; 2 Faculty of Education, Universiti Teknologi MARA, Puncak Alam, Selangor Malaysia; 3 College of Computing, Informatics and Media, Universiti Teknologi MARA, Shah Alam, Selangor Malaysia; 4 College of Creative Arts, Universiti Teknologi MARA, Puncak Alam, Selangor Malaysia; 5 Faculty of Business and Management, Universiti Teknologi MARA, Puncak Alam, Selangor Malaysia

**Keywords:** Education, Information systems and information technology

## Abstract

Higher education students are frequently required to assess lecturers with a convenient, fast, and anonymous learning management system. Following the coronavirus disease 2019 (COVID-19) pandemic outbreak, Universiti Teknologi MARA Malaysia (UiTM) adopted a remote teaching and learning approach. This study examined how lecturers’ professionalism, course impression, and facilitating conditions at UiTM affected undergraduate and graduate students’ remote learning pre- and mid-pandemic. The higher prediction accuracy of the model demonstrated that students’ remote learning activities were highly related to lecturers’ professionalism, course impression, and facilitating conditions. The structural model demonstrated that the *t*-statistics of all measurement variables were significant at 1%. The strongest predictor of students’ enjoyment of remote learning pre- and mid-pandemic was lecturers’ professionalism. In the importance-performance matrix, lecturers’ professionalism was in the quadrant for ‘keep up the good work’. Facilitating conditions and course impression did not require further improvement even during the pandemic. The influence of remote learning was demonstrated in the students’ graduation rates and grades. The results also presented theoretical and practical implications for the UiTM hybrid learning plan post-pandemic.

## Introduction

The term ‘remote learning’ is frequently used interchangeably with ‘distance learning’, ‘electronic (e)-learning’, and ‘online learning’. In remote learning, students are physically distant from their instructors and require internet-connected digital devices, such as laptops, tablets, desktop computers, and smartphones. Remote learning is delivered synchronously (lecturers and students are online at the same time) or asynchronously (lecturers and students are online at different times and locations if internet access is poor for either party) (Nketekete et al., 2021; Syahruddin et al., 2021). There are various means of conveying remote learning, which range from traditional take-home materials (books) to modern online and digital resources. Mobile phones, television, radio, and online tutors are all viable options that enable remote learning (Muñoz-Najar et al., 2021). University students are encouraged to undertake remote learning due to the potential benefits, which include greater learning flexibility, enhanced learning experience, learning personalisation, improved access to distance learning resources (Kamble et al., 2021), and support for higher student enrolment (Morris et al., 2019).

On March 18, 2020, the Malaysian government enforced the Movement Control Order (MCO) to prevent coronavirus disease 2019 (COVID-19) spread nationwide. During the MCO, all higher learning institutions were closed, and activities of learning and teaching shifted online. Thus, Universiti Teknologi MARA (UiTM), which is the largest Malaysian public university with 185,400 active students and 8,958 lecturers from 27 faculties and 27 UiTM branch campuses nationwide, was compelled to transition from in-person to remote learning.

During the pandemic, academic activities involved both asynchronous and synchronous remote learning. Students with low internet connectivity received learning materials via e-mail, while those with good internet connectivity used Google Meet or Webex for teaching and learning. Students with slow internet connections were given consideration in synchronous sessions, where sessions were flexible and could be moved to another scheduled time to avoid scheduling conflicts with other online sessions. Furthermore, lectures could be recorded and shared on Google Drive. Moreover, students could conduct self-paced asynchronous learning activities on the UiTM e-learning platform UFUTURE.

Nevertheless, the rapid transition from in-person to entirely online learning during the pandemic significantly challenged both instructors and students. One of the most challenging aspects of designing a good learning environment and student activities was developing online learning methods and courses that accommodated traditional methods (Amin et al., 2020). For example, Indian universities experienced a lack of facilities, instructional resources, and academic staff skilled in information technology (IT) staff. These deficiencies were expected to persist after the COVID-19 pandemic (Kaup et al., 2020). In Romania, students were predicted to demonstrate high perceived e-learning efficacy based on the internet connectivity availability in their homes and the university online learning infrastructure (Roman & Plopeanu, 2021). In Pakistan, societal views were associated with the shift to online learning. Students’ families conveyed irrefutable doubts or concerns about computers and networks, where the poorest families were unable to afford online learning devices. When parents and children are required to study or work from home, an unpleasant study and work environment can render work or learning increasingly challenging (Maqsood et al., 2021).

Students’ evaluation of teaching is prevalent in higher education. Students frequently evaluate their lecturers toward class completion or after the last semester class session. Student evaluations can provide lecturers with constructive feedback, which would improve the teaching methods of specific academic programme courses. Furthermore, such evaluations provide feedback on course instruction effectiveness, which is composed of delivery, substance, professionalism, assessment, grading, and innovation. Thus, most educational experts consider student evaluations an accurate, valid, reliable, and beneficial assessment tool (Darwin, 2017; North et al., 2018).

Before the pandemic, teaching and learning was mainly conducted in-person. A blended approach was used by lecturers, which included a learning management system (LMS) to provide lecture content, create topic discussion forums, and design coursework assessments tests and quizzes. Students from engineering, clinical, and arts courses participated in blended learning by attending formal lectures, working in labs or workshops, and participating in forum discussions online. At the end of the semester, students evaluated how well their lecturers taught, their lecturers’ professionalism, and what they had learned from the course.

Despite numerous studies on distance learning quality, satisfaction, and success (Azlan et al., 2020; Choi et al., 2021), Malaysian higher education research on measuring remote learning acceptance considering learning activities (LA), and lecturer professionalism (LP) is scarce. Thus, this study examined UiTM students’ remote learning acceptance through LP, course impression (CI), and facilitating conditions (FC) pre- and mid-COVID-19 outbreak.

## Materials

### Study hypotheses

The term ‘profession’ refers to a job that requires much expertise, focus on detail, and observance of recognised scientific and educational principles. The term ‘professionalism’ refers to a professional’s commitment to self-improvement. The LP is required to yield excellent performances that gratify all stakeholders, which include students, parents, and wider society (Hidayati & Siswati, 2018). As professionals, lecturers are required to possess numerous competencies to conduct their tasks appropriately, namely subject matter knowledge and skills; cognisance of their students’ knowledge, personality, and skills; and professional growth (Wiranto & Slameto, 2021). Vayre and Vonthron (2017) suggested that continuous support from lecturers and professors may aid students’ remote learning success. Shankar, Gowtham and Surekha (2020) reported that teachers’ professional and personal development is vital to enrich students’ learning experience. Creative and experienced lecturers design the intended LA based on current knowledge, content and delivery quality, and IT skills to increase student gratification (Almusharraf & Khahro, 2020; Wiranto & Slameto, 2021). Lecturers’ attitudes and competencies are important to develop positive student behaviours in technology-mediated distance learning (Rudhumbu, 2021). Furthermore, LP and infrastructure and facility quality influence alumni satisfaction with higher education institution learning (Azizan et al., 2022; Wiranto & Slameto, 2021). Thus, the following hypotheses (H) were proposed:

H1: The LP positively influences CI.

H2: The LP positively influences FC.

H3: The LP positively influences LA.

James et al. (2022) reported that meaningful online learning requires good pedagogical strategies, resources, and technology. Universities with a suitable IT infrastructure would enable greater student engagement and more effective study, as such an infrastructure establishes an appealing and conducive learning environment (Wiranto & Slameto, 2021). Conversely, students with poor home internet connections are likely to experience online learning issues (Kamble et al., 2021). Zakharov and Maybee (2019) stated that students with poor home internet connectivity were 10% more likely to study online ineffectively. Teo, Kim and Jiang (2020) demonstrated that the proactive role in constructing a solid learning network and facility infrastructure in Korea significantly influenced the national e-learning platform effectiveness.

Moreover, distance education efficacy can be affected by students’ traits, such as ethnicity, gender, and active participation in various e-learning community platforms (Alasmari, 2021). For example, remote learning efficacy was influenced and improved by dedicated LMS support platforms, such as Google Meet and Zoom, thus suggesting that university remote learning platforms were typically satisfactorily adapted to crises, which included the COVID-19 pandemic. Therefore, such platforms established safe, secure, and agreeable e-learning environments in developed and developing countries (Roman & Plopeanu, 2021). Based on the aforementioned information, the following hypotheses were proposed:

H4: The CI positively influences LA.

H5: The FC positively influence LA.

### Study model

**Fig. 1:**
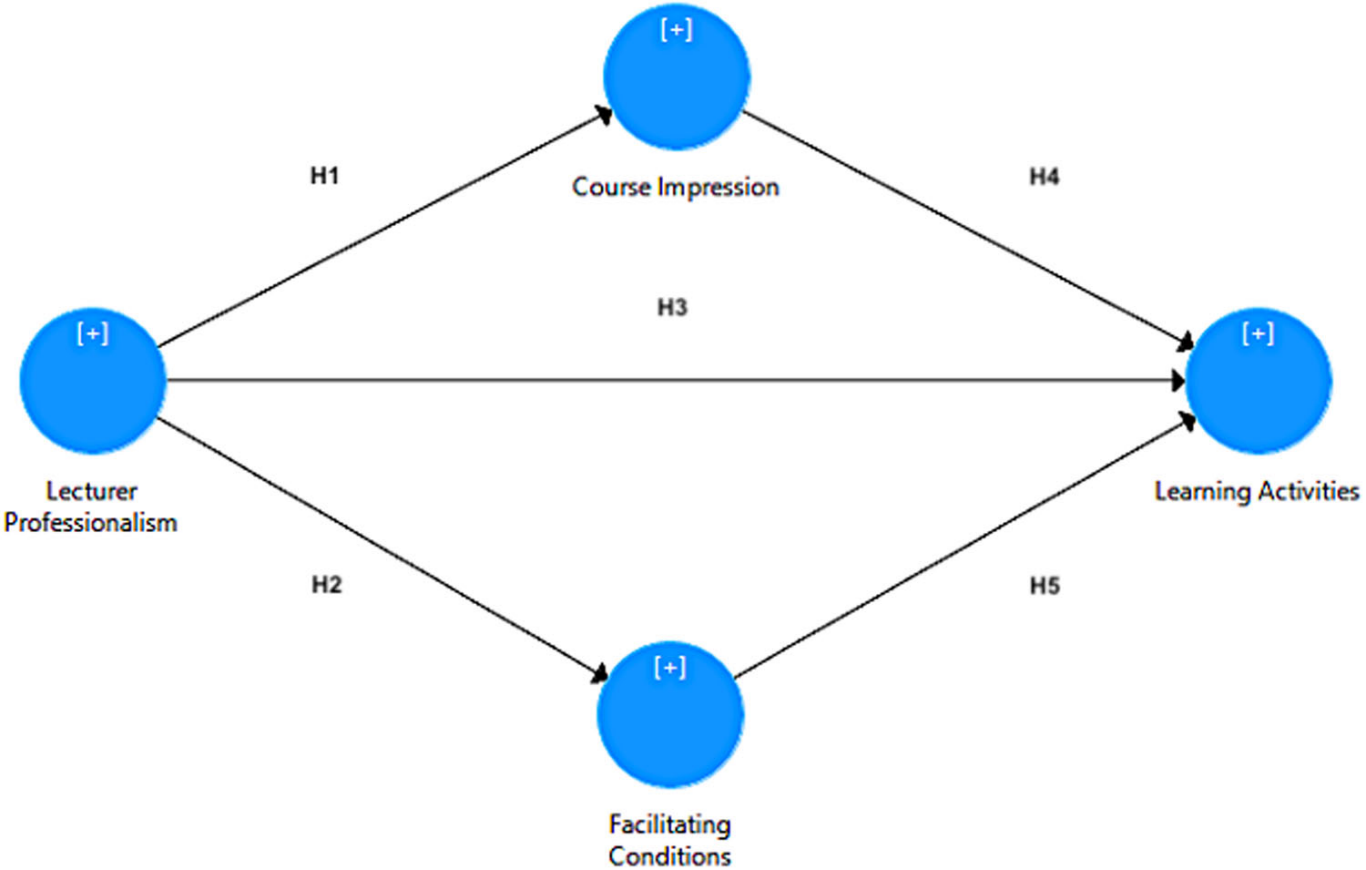
Proposed remote learning acceptance model (drawn in partial least squares structural equation modeling).

The model design was inspired by Danielson’s (2007) framework for teaching, which is a set of teaching performance indicators or standards measured by four-level evaluation scales or rubrics: content and pedagogy planning and preparation, classroom environment, instruction, and professional responsibilities. The framework was modified to accommodate the context of Malaysian higher education, and included CI and FC as the new measurement variables. Figure 1 demonstrates the three independent variables (LP, CI, and FC) and one dependent variable (LA) proposed in this study.

The LP refers to self-awareness, commitment, professionalism, and leadership as desirable traits to improve personal and professional competence development throughout academic fields by satisfying all stakeholders, which are primarily students (Hernandez et al., 2021; Hidayati & Siswati, 2018). The CI refers to the learners’ expectations or self-reflection of knowledge gained, field relatedness, learning ability, and remote learning confidence level (Almaiah et al., 2016; Yuan et al., 2021). The FC refers to the student’s perception of available IT resources, tools, and support, which includes the related devices and internet data plans (de Witte et al., 2021; Patricia Aguilera-Hermida, 2020) used to achieve the planned learning objectives and outcomes. Lastly, LA refers to the degree to which learning becomes an enjoyable and important experience for students (Rizun & Strzelecki, 2020; Syahruddin et al., 2021). A positive relationship between LP, CI, and FC creates an enjoyable LA. Thus, students will enjoy their LA if they have a good first impression, good learning environment, and professional lecturers, which affect their perception and acceptance of remote learning.

## Methods

### Research design

The model in this quantitative study was converted into academic policy for lecturer teaching evaluation and has been applied since September 2010 (see Supplementary Information).

### Instrument

The online evaluation form contained four sections with 24 questions measured on a four-point forced Likert scale ranging from ‘strongly disagree’ (1) to ‘strongly agree’ (4). Section A contained four questions on the overall CI, Section B contained seven LP questions, Section C contained 11 LA questions, and Section D contained two learning infrastructure questions.

### Unit of analysis or data sources and data collection

The respondents were UiTM undergraduate and postgraduate students. Every learning semester, the students are required to evaluate their lecturers within six weeks (from week 11 to week 16) using Student Feedback Online (SuFO), which can be accessed through the LMS UFUTURE (https://ufuture.uitm.edu.my/home). Typically, one student will evaluate at least five lecturers per semester based on the course credit hours registered. For example, a student who registers for seven courses (out of the 5000 courses are offered) involving 19 credit hours per semester must evaluate one lecturer per course. Lecturers can view their evaluation scores one week after the student examination results have been released.

### Data analysis

The SuFO data for the October 2019 to February 2020 semester (pre-pandemic) and the March to August 2020 semester (mid-pandemic) were extracted. The descriptive data containing the respondents’ profiles were analysed in the Statistical Package for Social Sciences (SPSS) software. The inferential data were analysed in the partial least squares structural equation modeling (PLS-SEM) programme.

## Results

### Descriptive analysis

A total of 92,752 students rated 7216 lecturers pre-pandemic, and 95,747 students evaluated 6647 lecturers mid-pandemic. The data underwent outlier checking to ensure data reliability and validity. The final pre- and mid-pandemic datasets contained 194,559 and 221,366 responses, respectively, which could be used for data analysis. Unengaged responses with a standard deviation value of <0.30 were discarded, as the respondents answered with the same scale for all questions; hence, these responses were not useful for data analysis (Lowry & Gaskin, 2014).

Table 1 presents the respondents’ demographic profile. Up to 95.4% (81,938) of undergraduates and 4.6% (3951) of postgraduates evaluated their lecturers pre-pandemic, while 95.1% (65,045) of undergraduates and 4.9% (3361) of postgraduates evaluated their lecturers mid-pandemic in three study clusters (science and technology, social science and humanities, and business and management). Up to 69.0% (1472) of undergraduate and 31.0% (661) of postgraduate programme courses were evaluated pre-pandemic, while 68.3% (1375) of undergraduate and 31.7% (639) of postgraduate programme courses were evaluated mid-pandemic.

**Table 1:** Respondents’ profile.

	Undergraduate *N* (%) *(pre-pandemic)*	Postgraduate *N* (%) *(pre-pandemic)*	Undergraduate *N* (%) *(mid-pandemic)*	Postgraduate *N* (%) *(mid-pandemic)*
Demographic/level				
Faculties/Academic Centres’ Clusters:				
▪ Science & Technology (10)	1536 (1.9)	1360 (34.4)	1351 (2.1)	1080 (32.1)
▪ Social Science & Humanities (9)	3295 (4.0)	855 (21.6)	2712 (4.2)	727 (21.6)
▪ Business & Management (6)	148 (0.2)	1343 (34.0)	421 (0.6)	1136 (33.8)
Branch Campuses	76,959 (93.9)	393 (9.9)	60,561 (93.1)	418 (12.4)
Total *N* (%)	81,938 (95.4)	3951 (4.6)	65,045 (95.1)	3361 (4.9)
Evaluated courses				
Total *N* (%)	1472 (69.0)	661 (31.0)	1375 (68.3)	639 (31.7)

### Measurement model

The final pre- and mid-pandemic datasets were analysed in PLS-SEM, which measures the evaluation data from large sample sizes by evaluating two models: path measurement and path analysis (Hayes et al., 2017). Table 2 demonstrates that assessing the measurement model validity for both datasets required outer loadings, composite reliability (CR), average variance extracted (AVE), and discriminant validity. Multi-collinearity assessment determined that the independent variable tolerance levels ranged from 1.4 to 2.8, which were below the critical threshold variance inflation factor (VIF) of 5, thus indicating that there were no issues (Hair et al., 2011). For convergent validity, the LP, CI, FC, and LA outer loadings exceeded the critical value of 0.7 in both datasets, excluding the LP3 and LA8 indicators, which were subsequently removed. The indicators demonstrated high reliability (Hair et al., 2017). Furthermore, the CR of the measuring variables (LP, CI, FC, and LA) exceeded the required threshold of 0.7, and the AVE values exceeded the minimum threshold of 0.5, which indicated that both datasets had high convergent validity (Hair et al., 2017). Therefore, the mid-pandemic dataset reflected higher outer loadings of each construct indicator, CR, and AVE score.

**Table 2:** Convergent validity of measurement model.

Latent variable	Item	Outer loadings *(pre)*	Outer loadings *(mid)*	VIF *(pre)*	VIF *(mid)*	AVE *(pre)*	AVE *(mid)*	CR *(pre)*	CR *(mid)*
Lecturer professionalism (LP)	LP1: The lecturer completes the scheduled hours of instruction.	0.744	0.781	1.705	1.952	0.606	0.658	0.902	0.920
	LP2: The lecturer is ever ready to provide academic guidance to students.	0.792	0.824	1.922	2.227				
	LP4: The lecturer is approachable.	0.789	0.817	2.002	2.361				
	LP5: The lecturer is accessible for discussion.	0.788	0.826	1.984	2.417				
	LP6: The lecturer monitors student attendance.	0.746	0.779	1.672	1.877				
	LP7: Overall, the lecturer exhibits high professionalism.	0.809	0.837	1.995	2.303				
Course impression (CI)	CI1: I have increased my knowledge from taking the course.	0.799	0.824	1.625	1.815	0.622	0.655	0.868	0.883
	CI2: The course content is related to my field of study.	0.737	0.756	1.471	1.560				
	CI3: The method of assessments in this course has enhanced my learning ability.	0.832	0.852	1.815	2.026				
	CI4: My confidence level in this course has increased.	0.784	0.802	1.573	1.715				
Facilitating conditions (FC)	FC1: The equipment space for teaching and learning is conducive.	0.935	0.946	2.281	2.827	0.875	0.902	0.933	0.948
	FC2: The teaching and learning equipments are adequate and functioning.	0.936	0.953	2.281	2.827				
Learning activities (LA)	LA1: The lecturer explains the course content.	0.776	0.802	2.256	2.589	0.610	0.651	0.940	0.949
	LA2: The lecturer explains the outcomes of the course.	0.770	0.798	2.243	2.560				
	LA3: The lecturer explains the methods of assessment for the course.	0.787	0.811	2.291	2.584				
	LA4: The lecturer teaches according to plan.	0.766	0.800	2.068	2.347				
	LA5: The lecturer actively involves students in the learning process.	0.782	0.810	2.172	2.442				
	LA6: The lecturer creates an environment for students to ask questions and offer opinions.	0.771	0.799	2.126	2.379				
	LA7: The lecturer delivers the content interestingly.	0.771	0.792	2.172	2.369				
	LA9: The lecturer provides feedback for each assessment/assignments/tests/projects.	0.788	0.803	2.220	2.396				
	LA10: The lecturer helps students master the learning content.	0.798	0.829	2.336	2.721				
	LA11: Overall, I enjoyed the teaching style of this lecturer.	0.797	0.822	2.377	2.694				

Based on the lack of sensitivity, discriminant validity was assessed with the heterotrait-monotrait ratio (HTMT) criteria rather than Fornell-Larcker criterion and cross-loadings. All HTMT values between constructs in the pre-and mid-pandemic models were less than the cut-off point of 0.90 (see Table 3), which demonstrated sufficient discriminant validity (Henseler et al., 2014).

**Table 3:** Discriminant validity of measurement model.

Latent variable	CI	FC	LA
*Pre-pandemic*			
FC	0.339		
LA	0.639	0.456	
LP	0.620	0.406	0.900
*Mid-pandemic*			
FC	0.348		
LA	0.647	0.435	
LP	0.617	0.386	0.904

### Structural model

The structural model predictive relevancy pre- and mid-pandemic was analysed. The LA denoted a strong coefficient of determination (*R*^2^) of 0.692 and 0.722 in the pre- and mid-pandemic models, respectively, which indicated that this dependent or outcome variable was described by approximately 50% of its variance by LP, CI, and FC. For effect sizes (*f*^*2*^), LP had a large effect on CI but a medium effect on FC. The LP had a large effect on LA, but CI and FC demonstrated small effects on LA in both models (Chin, 1998). Regarding significance, Hair et al. (2017) proposed that a *t*-value of at least 2.53 when the *p*-value is ≤0.01 (1%) indicated significance. The structural model was estimated using complete PLS bootstrapping with 5,000 subsamples. All tested hypotheses were supported, as all measuring variables were positive and significant at the level of 0.01 (see Table 4).

**Table 4:** Path analysis & hypothesis testing.

Outcome variable/coefficients (*R*^2^)	Hypothesis/path	*β*-Value	*f*^2^-value/size	*t*-value	Decision
*Pre-pandemic*					
LA (*R*^2^ = 0.692)	H1: LP → CI	0.519	0.368 (large)	238.987***	Supported
	H2: LP →FC	0.351	0.140 (medium)	128.105***	Supported
	H3: LP → LA	0.682	1.033 (large)	343.034***	Supported
	H4: CI → LA	0.164	0.063 (small)	81.336***	Supported
	H5: FC → LA	0.121	0.041 (small)	81.912***	Supported
*Mid-pandemic*					
LA (*R*^2^ = 0.722)	H1: LP → CI	0.532	0.394 (large)	260.694***	Supported
	H2: LP → FC	0.345	0.135 (medium)	146.913***	Supported
	H3: LP → LA	0.706	1.220 (large)	419.021***	Supported
	H4: CI → LA	0.164	0.068 (small)	94.651***	Supported
	H5: FC → LA	0.106	0.035 (small)	78.806***	Supported

***Significant at 0.01 level.

In both models, the strongest effect was LP on LA, which confirmed H3, followed by the effect of LP on CI (H1), LP on FC (H2), CI on LA (H4), and FC on LA (H5), which confirmed these hypotheses. The mid-pandemic dataset revealed higher scores for model prediction (*R*^2^), significance (*t*-values), and effect sizes (*f*^*2*^ values).

### Extended analysis

The importance-performance map analysis (IPMA) is an extended PLS procedure to measure LA as the target variable following the structural model evaluation. In this study, the IPMA objective was to identify variables that were important to the target construct (LA) but that underperformed, wherein managerial actions could be focused on that specific construct for improvement (Hair et al., 2017). Table 5 presents the IPMA results, where the highest total effect score was for LP (0.810) at the performance level 84.54 in the pre-pandemic model and for LP (0.830) at the performance level 85.45 in the mid-pandemic model. The results demonstrated an increase in LP from 84.5% pre-pandemic to 85.5% mid-pandemic. Conversely, the FC decreased from 78.8% pre-pandemic to 73.1% mid-pandemic.

**Table 5:** Importance-performance map analysis results.

Latent variable	Importance (total effect)	Performance (%)
*Pre-pandemic*		
LP	0.810	84.54
CI	0.162	82.14
FC	0.091	78.80
*Mid-pandemic*		
LP	0.830	85.45
CI	0.165	82.02
FC	0.078	73.13

**Fig. 2:**
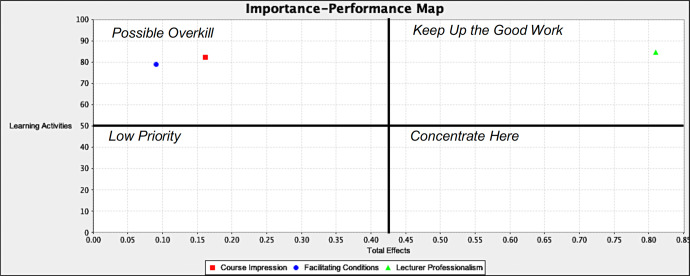
Increased remote learning activities pre-pandemic.

**Fig. 3:**
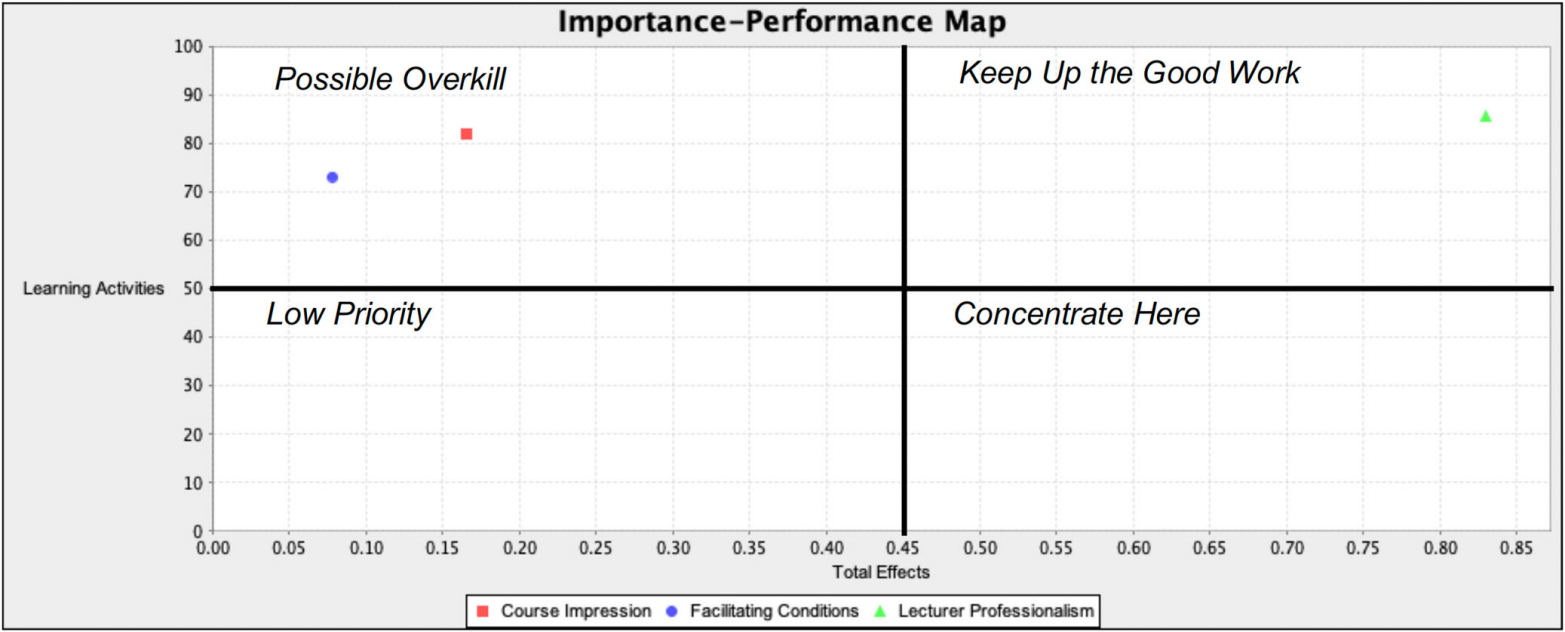
Increased remote learning activities mid-pandemic.

The LP was in the ‘keep up the good work’ quadrant, which confirmed the significant result on the positive effect of LP on LA (see Figs. 2 and 3). The increased LP predicted increased students’ remote learning activity in pre- (Fig. 2) and even mid-pandemic (Fig. 3) academic sessions.

## Discussion

The year 2020 was challenging for UiTM students, as they were unable to learn in-person. Nevertheless, the lecturers demonstrated unequivocal commitment to ensuring that the students enjoyed distance learning and were able to share experiences. The lecturers were committed to remote teaching and constantly enabled students’ assessments and assignments throughout the semester, which indicated increased LP.

The findings were consistent with those of previous studies, where CI positively affected LA pre- and mid-pandemic (Alasmari, 2021; Hamadi et al., 2021; James et al., 2022). As students learn more about remote learning and its contents, they feel happier about their decision to use it in learning (Teo et al., 2020). Furthermore, the LA pre- and mid-pandemic positively affected FC. This finding paralleled that of Roman and Plopeanu (2021), who reported that learning infrastructure aided and supported distance education in Rome universities and that good IT infrastructure aided online learning by Malaysian university students (Munir et al., 2021). At UiTM, students from the B40 household group (income <RM2500 per month) were hampered by poor internet access, insufficient data plans, low-performing laptops, and unconducive learning environments. These issues contributed to their lack of motivation, tiredness, and learning passivity, which could worsen, cause depression and anxiety, and eventually lead to study postponement.

The LP significantly affected the CI and LA pre- and mid-pandemic, which corresponded to Vayre and Vonthron’s (2017) study. Kara, Tanui and Kalai (2016) confirmed that LP and the quality of teaching methods, the Internet, and learning module accessibility contributed to a high level of educational services and student satisfaction in Kenyan state universities. Distance education and e-learning success is determined by reliable online aid from teachers and professors (Almusharraf & Khahro, 2020; Roman & Plopeanu, 2021; Vayre & Vonthron, 2017; Wiranto & Slameto, 2021) and lecturers’ technological self-efficacy (Rudhumbu, 2021), which supported the significant result of LP on LA. Improving e-learning facilities and services can enhance LP (Alasmari, 2021; Wiranto & Slameto, 2021).

Essentially, conventional learning was converted to a new learning norm that involved more student-centred LA. A flipped classroom engages students more instead of requiring them to wait for lecture notes and assignments. Flipped classrooms enhance knowledge transfer and class time efficiency (Chiquito et al., 2020). Students acquire key concepts and terminologies by reading or viewing recorded lecture videos on their mobile devices before class, which facilitates flexibility and self-paced learning (Howell, 2021). The Universal Design of Learning requires lecturers to design self-instructional materials (SIMs) and learning processes based on students’ capability and accessibility using low- and high-tech devices and connectivity. Thus, the UiTM lecturers prepared materials in digital formats [portable document format (PDF), PowerPoint Presentation (PPT), or massive open online course (MOOC)] and shared the SIMs on UFUTURE and social networking platforms, such as WhatsApp, Telegram, and Facebook group pages. The rapidly prepared SIMs enabled self-efficacy learning, where students’ understanding was subsequently reinforced in discussion or tutorial sessions. Learning flexibility was promoted when lecturers conducted video-based synchronous sessions in their first one-hour lectures before enabling learning personalisation in tutorials via WhatsApp and Telegram. Students mainly applied problem-based learning to group work and peer learning. These approaches were consistent with that of Vázquez-Cano and Díez-Arcón (2021), who demonstrated that remote learning university students used Facebook groups as an efficient learning tool. The online learning efficacy increased learner satisfaction, specifically regarding productivity and motivation.

de Brún et al. (2022) reported that lecturers at a large urban university in Ireland evaluated graduate students with the appropriate tools and techniques. The approach included exemplars, rubrics, explicit scoring, and feedback criteria in online assessment to provide constructive feedback and improve online assessment validity and accuracy. Student-centred online assessment via LMS can enhance personal and professional growth, deep learning, and transferable skills, such as critical thinking (Nelwati et al., 2018).

Under UiTM remote learning protocols, learning assessment was customised without affecting learning outcome achievement, specifically for exam-based courses, by modifying assessment methods, task number, duration, and parameters, and the application process. For example, an online test assessment could measure more than one course learning outcome, where there could not be more than four course assessment tasks. Furthermore, assessment durations and processes for exam-based courses were rescheduled throughout the semester rather than during exam weeks. Typically, online assessments were applied in UFUTURE and aligned with course learning outcomes. Specifically, students’ learning objective mastery or progress was measured with online formative assessment. Such assessment provided feedback on student learning improvement and contributed to the final grade. At the end of the learning semester, the students underwent a three-week online summative assessment to ensure that they had achieved the expected learning outcomes. These online formative and summative assessments were conducted in two situations:

- Synchronous assessment, which were implemented if students had good internet access.
- Asynchronous assessment, which allowed lecturers to plan and design assessments that could be conducted within a set period using different sets of questions but using the same level of difficulty.

The findings indicated that higher LP enhanced the students’ remote learning enjoyment. The significance test and IPMA results were compared with actual student achievement data on graduate-on-time (GOT) and the cumulative grade point average (CGPA). Remote learning enjoyment contributed to a slight decrease in the GOT rate of 1.5% among UiTM undergraduates in 2020 as compared to the pre-pandemic period in 2019. Similarly, the 2020 GOT among UiTM postgraduates who undertook Master’s degrees by coursework decreased slightly by 1.7% compared to the previous year. When overall academic performance during the pandemic year was evaluated using the CGPA, 83.1% of undergraduates achieved good and excellent CGPAs between 3.00 and 4.00. Comparatively, 48.7% of postgraduates in coursework programmes obtained good and exceptional CGPAs between 3.00 and 4.00.

The results demonstrated that the UiTM students obtained consistent GOT rates and CGPAs, which confirmed their satisfaction with the remote learning experience during the pandemic. The findings were supported by Refae et al. (2021), who reported that using new technologies and digital resources could aid university student performance improvement when they learned from home. Rajadurai et al. (2018) stated that distance learning technologies, course effectiveness and quality, and digital resource usefulness were some of the most important factors for improving student performance.

While increased LP benefited LA, criticisms involving excessive assignments, no feedback on assignments, inadequately experienced lecturers designing online lessons, and students encountering difficulty in understanding presentation platforms and methods were recorded. The lecturers were also unaware that the students experienced learning and accessibility limitations. Hebebci et al. (2020) stated that technological unpreparedness was a major disadvantage of distance learning. In India, Nambiar (2020) reported that the main online classroom concerns were technical issues, such as slow internet connections, bad video quality, and difficulty logging in to different courses. Additionally, there were a few instances of mental health issues and unsupportive families.

The UiTM lecturers were entirely new to the remote teaching approach and required time for adjustment. Nonetheless, they participated in the numerous weekly online comprehensive training sessions or courses offered by the university to equip themselves with the required IT knowledge and skills in their respective teaching fields. The training sessions and courses ranged from content development to online coursework assessment. Despite UFUTURE being the central LMS, the university did not restrict e-learning platforms to improve students’ learning experiences through numerous delivery techniques to achieve course learning objectives and outcomes. Thus, lecturers were able to use other LMS (Google Classroom, Microsoft LMS365, and Blackboard Learn) and the Zoom and Webex platforms for synchronous classes, and communicate and monitor students via social media platforms (Facebook and Telegram). This finding paralleled Irfan et al. (2020) qualitative case study, where LMS (Google Classroom and Edmodo) and video conferencing (Zoom and Skype) supported online learning during the pandemic in Indonesia and positively influenced teaching. Thus, technological quality and system content substantially influence students’ satisfaction with LMS and perception of its usefulness (Nguyen, 2021).

## Conclusion and implications

This study evaluated UiTM students’ feedback regarding remote learning before and throughout the pandemic under the MCO. The students reported increased enjoyment of remote LA presented by dedicated and professional lecturers. The students were satisfied with their learning experience, which was confirmed by the steady GOT rates and satisfactory CGPAs. The findings confirmed that the students were prepared for remote learning and were good remote learners. The students’ academic achievements were sustained without a reduction in GOT targets of 80% during the pandemic year, despite the immediate change in learning mode that forced them to learn online from home. The students became more independent, self-directed learners, and active social media users in forum discussions and tutorials. Personalised learning was promoted and granted greater flexibility for examinations and assessment deadlines. Thus, the students changed how they learned, worked, and interacted with others to complete their studies on time with good results. Integrating LP with CI and FC demonstrated that the dependent variable LA denoted higher explanatory and predictive power, specifically during the pandemic.

To create enjoyable learning activities for UiTM students post-pandemic, policymakers should revise the current policy to empower LP through relevant training sessions and webinars on ethical design and digital content use, effective online communication, value-centred learning, collaborative teaching, alternative assessment, and psychological well-being learning management. Furthermore, university management should allocate a more substantial budget to improve UFUTURE as the primary e-learning platform with current assessment and pedagogical tools to enhance usage, student and lecturer confidence, and learning motivation post-pandemic. The management should emphasise CI and FC less, as they are less important when measuring students’ enjoyment of remote learning pre- and mid-pandemic.

This study established and validated a simplified model of the determinants of enjoyable remote LA among UiTM undergraduates and postgraduates with a large dataset of pre-and mid-pandemic academic sessions and significant findings. Thus, LP to promote enjoyable student LA at UiTM was an important determinant of remote learning acceptance pre- and mid-pandemic. Determining the factors that can increase LP is critical to continuously provide an enjoyable and meaningful learning experience to UiTM students. Nevertheless, B40 students were worried and dissatisfied with their required computers and networks for learning during the outbreak.

Malaysia has made significant progress in controlling the spread of COVID-19 by implementing strict measures such as lockdowns, travel restrictions, and widespread testing and vaccination programmes. These measures have resulted in a decrease in the number of new cases and deaths. Therefore, as the country moves towards the post-pandemic era, it is important for UiTM to carefully adjust its implementation plan for hybrid learning. This should be done to accommodate students’ emotional and learning needs to ensure that they will GOT with the necessary skills and competencies required for future jobs. Thus, the findings presented practical implications in the form of the following recommendations to UiTM for a better remote learning experience:

- University policymakers can revise the existing teaching and learning policy to empower LP through 360° teaching evaluation by students, peers or colleagues, and self-reflection.
- The revised policy should acknowledge students’ remote learning accessibility. Accessibility does not merely involve internet or computer access, as family conditions can influence levels of concentration and learning material access. Furthermore, online classes must emphasise tolerance, adaptability, and communication.
- Faculties and state branches should assist in expanding students’ remote learning access by providing grants, equipment loans, and campus entrance to B40 students and students registered for practical, studio, and clinical-based courses.
- The revised policy can also guide professors or lecturers’ pedagogical and psychological skills and their related personalities, which may affect the relationship with students in many ways.
- Remote learning does not solely refer to course content, delivery, and assessment. Emergency remote learning during the pandemic necessitated a high degree of self-discipline, as students were required to manage their learning processes at home. Lecturers must continuously promote a positive attitude throughout the semester, while students must remember and be reminded to manage their workload systematically to achieve the expected course learning outcomes. To monitor learning, both lecturers and students can benefit from social platforms (WhatsApp and Telegram).
- Instructional support may be presented as constructive feedback, responsive communication, forums, and discussion through UFUTURE and the relevant learning resources and activities. Additionally, student communities can be fostered by introducing peer connections or learning buddies.
- Establishing remote learning communities would guarantee staff and student wellness and facilitate social and pastoral care. Digital access and literacy can be improved by promoting engagement and by lecturers who are flexible, kind, and patient.

Given that the study was conducted during the COVID-19 pandemic, the students’ perceptions could have been influenced by fear, stress, uncertainty, depression, distractions, and financial hardships, which are both limitations and opportunities. Students would be able to express themselves better if surveyed during the transition from pandemic to post-pandemic rather than later. The results should be considered with caution due to the aforementioned limitations. Future studies can consider other possible factors, such as personal attributes and competence, emotional intelligence, positive attitude, self-efficacy, learning satisfaction, and motivation, to extend the study model and evaluation instrument in forecasting university students’ enjoyable remote learning that eventually affects their overall learning outcomes and performance.

## Data Availability

The datasets generated during and/or during the current study are not publicly available due to the confidentiality of the respondents’ information but are available from the corresponding author upon reasonable request for academic purposes only.

## Informed consents

Informed consent was obtained from all students before the data was collected. We informed each student of their rights and to safeguard their personal information.
